# New insights into pulmonary arterial hypertension: interaction between PANoptosis and perivascular inflammatory responses

**DOI:** 10.1007/s10495-025-02086-0

**Published:** 2025-02-20

**Authors:** Xianli Su, Yinhui Sun, Aiguo Dai

**Affiliations:** 1 College of Clinical Medicine, Hunan University of Chinese Medicine, Changsha, Hunan 410208 People’s Republic of China; 2 Hunan Provincial Key Laboratory of Vascular Biology and Translational Medicine, Changsha, Hunan 410208 People’s Republic of China; 3 School of Integrated Chinese and Western Medicine, Hunan University of Chinese Medicine, Changsha, Hunan 410208 People’s Republic of China; 4 Department of Respiratory Medicine, School of Medicine, Changsha, Hunan 410021 People’s Republic of China; 5 Department of Respiratory Medicine, The First Affiliated Hospital of Hunan University of Chinese Medicine, Changsha, Hunan 410021 People’s Republic of China

**Keywords:** Pulmonary artery hypertension, PANoptosis, Inflammation, Apoptosis, Pyroptosis, Necroptosis

## Abstract

Pulmonary arterial hypertension (PAH) is a heterogeneous disease characterized by various etiologies, with pulmonary vascular remodeling recognized as a main pathological change. Currently, it is widely accepted that vascular remodeling is closely associated with abnormal pulmonary vascular cell death and perivascular inflammation. The simultaneous activation of various pulmonary vascular cell death leads to immune cell adhesion and inflammatory mediator releases; And in turn, the inflammatory response may also trigger cell death and jointly promote the progression of vascular remodeling. Recently, PANoptosis has been identified as a phenomenon that describes the simultaneous activation and interaction of multiple forms of programmed cell death (PCD). Therefore, the relationship between PANoptosis and inflammation in PAH warrants further investigation. This review examines the mechanisms underlying apoptosis, necroptosis, pyroptosis, and inflammatory responses in PAH, with a focus on PANoptosis and its interactions with inflammation. And it aims to elucidate the significance of this emerging form of cell death and inflammation in the pathophysiology of PAH and to explore its potential as a therapeutic target.

## Introduction

Pulmonary arterial hypertension (PAH) is a complex and serious public health problem, with therapeutic effects and prognosis are less than ideal. A clinical study involving 1193 subjects demonstrated that the average survival time for Group 1 PAH patients was 4.6 years, whereas for the other groups was 1.3–3.2 years [1, 2]. More concerning is the overall prevalence of PAH in China, which has reached 3.8%, with approximately 4.3% classified as Group 2 [3]. Therefore, the investigation of PAH pathogenesis is particularly urgent to achieve the goals of early diagnosis and precise treatment. The 2022 European Society of Cardiology/European Respiratory Society (ESC/ERS) guidelines divide PAH into five groups in accordance with different pathogeneses [4], but vascular remodeling has always been considered a common marker and major pathophysiological change in various PAH [5]. It is primarily characterized by the dysfunction of pulmonary vascular cells and the infiltration of peripheral inflammatory cells [6], with the dysfunction of pulmonary vascular cells closely related to programmed cell death (PCD) [7]. And it has been reported that excessive expression of cell death (such as pyroptosis, necroptosis, etc.) can trigger cytokine storms and inflammation [8, 9]. Therefore, it is believed that the two are not only parallel in PAH but also interact to aggravate PAH progression.

PCD is a cellular suicide mechanism to maintains homeostasis and triggers cell death by activating cascades of transcriptional and posttranslational protein modifications [10]. Apoptosis, necroptosis, and pyroptosis are the classic and well-defined death modes of PCD, and morphological characteristics are the main basis for distinguishing between these three modes of cell death. Apoptosis was first characterized in 1972 and is an indispensable way of cell death to maintain homeostasis [11]. Its morphological features include cell volume reduction, chromatin condensation, nuclear pyknosis, apoptosome formation and cytoskeletal disintegration [12]. And then new forms of cell death were constantly identified for the ongoing attention in this area of research. In 2001, a novel pro-inflammatory form of PCD, distinct from both apoptosis and necrosis, was discovered and officially named pyroptosis [13]. Pyroptosis is a soluble and inflammatory death mediated by the gasderm (GSDM) protein family [14], characterized by rapid rupture of plasma membrane and release of pro-inflammatory substances [15]. Necroptosis was first discovered and formally named by Degterev and Alexei in 2005 [16], which is a form of inflammatory cell death independent of caspases [17]. Its morphologies include the loss of plasma membrane integrity, swelling and deformation of organelles and release of cellular contents that promote inflammation [18]. Although differences exist in the morphological alterations among the three types of cell death, clear crosstalk is exhibited at the molecular level [19]. Additionally, Malireddi [20] discovered a highly interconnected cell death and named "PANoptosis" in 2019. The emergence of this new concept further confirms the crosstalk among different cell death. PANoptosis has been found to play a crucial role in many diseases since 2019, including infectious diseases, tumors, neurodegenerative diseases, and cardiovascular diseases [21]. PAH, a fatal cardiovascular disease with low survival rates and poor prognosis, is associated with cell death in pulmonary vascular cells, including apoptosis, necroptosis, pyroptosis, and autophagy [7, 15]. In summary, PANoptosis may be a important mechanism in PAH pathogenesis.

**Fig. 1:**
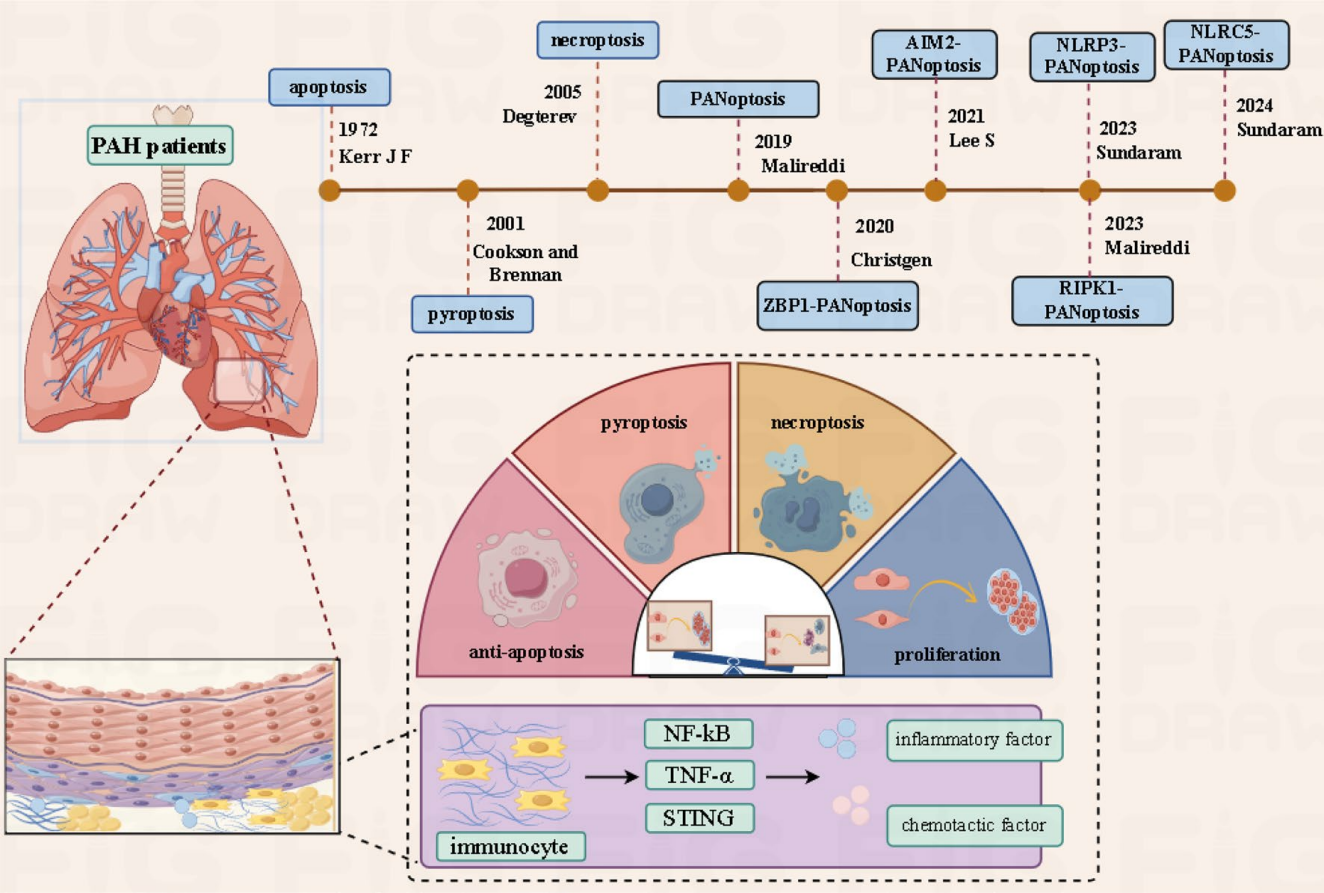
Pathophysiological mechanisms of PAH. Vascular remodeling is the critical pathophysiological mechanism in PAH, and is primarily composed of abnormal proliferation, death of pulmonary vascular cells, and peripheral vascular inflammation. The peripheral vasculature is primarily formed by various immune cells that release inflammatory mediators and chemokines via partially regulated factors. The abnormal death of pulmonary vascular cells mainly involves apoptosis resistance, pyroptosis, and necroptosis. Apoptosis was proposed in 1972, pyroptosis was officially named in 2001, and necroptosis was discovered in 2005. As of 2019, a total of five types of PANoptosis have been identified. This diagram was drawn by Figdraw (www.figdraw.com)

Infiltration of perivascular inflammation also aggravates pulmonary vascular resistance in PAH by affecting pulmonary hemodynamics and vascular remodeling [22]. As shown in Table 1, the expression levels of inflammatory factors vary among different subgroups of PAH, and some inflammatory factors are closely related to the prognosis of PAH. It has been reported that inflammatory factors and chemokines can induce PCD and aggravate the formation of vascular remodeling by activating upstream factors of multiple death pathways [22–24]. Existing evidence shows that some PCD (such as ferroptosis, apoptosis resistance, and pyroptosis)-mediated inflammatory responses can aggravate PAH [25–27]. Although there are few studies on the mechanisms of necroptosis [28, 29] and autophagy [30, 31] in PAH, they have been shown to trigger peripheral inflammation in other diseases. Thus, it is evident that some PCD have a causal relationship with inflammation in PAH. Furthermore, the fundamental characteristics of PANoptosis, which encompasses apoptosis, necroptosis, and pyroptosis, indicate that it may also interact with perivascular inflammation in PAH, further promoting vascular remodeling and hemodynamic changes (Fig. 1).

**Table 1:** Inflammatory factors involved in PAH

Category	Source	Target	PAH classification	Expression	Inflammatory pathways	Cell death
IL-1β	myeloid cells ([32])	PASMCs ([22, 32])	I ([33, 34]), II ([32]), III ([35–37]), IV ([38, 39])	↑	IL-1R1/MyD88,IL-1β/NF-κB/Snail ([40, 41])	pyroptosis ([42]), apoptosis ([43]) autophagy ([44])
IL-6	PAECs PASMCs fibroblastmacrophage T cell B cell mast cell dendritic cell ([45, 46])	PAECs ([47]) PASMCs ([48]) macrophage	I ([33]), II ([49, 50]), III ([51]), IV ([39])	↑	JAK/STAT JAK/Ras/Raf JAK/PI3K/AKT IL-6/NF-κB IL-6/MAPK/ERK IL-6/BMP IL-6/Notch IL-6/Fe ([45])	Apoptosis ([26]) Autophagy ([52, 53]) pyroptosis ([42])
IL-18	dendritic cell PASMCs Macrophage ([54])	PASMCs ([55])	I ([56]), III ([57])	↑	IL-18/IL-Bpa ([58])	Apoptosis ([59]) Pyroptosis ([42]) autophagy ([60])
MCP-1/CCL2	VECs VSMCs ([61])	monocyte macrophage PASMCs ([61])	I ([62]) III ([63]) IV ([64])	↑	CCL2/CCR2 ([63])	apoptosis, autophagy ([65, 66])
CCL5/RANTES	T cell NK cell PAECs macrophage ([67, 68])	T cell NK cell PASMCs PAECs ([68])	I ([68])	↑	CCL5/BMP ([69]) CCL5/CCR5 ([68])	apoptosis ([69])
CX3CL1/Fractalkine	PASMCs PAECs epithelial cell ([70, 71])	monocyte lymphocytePASMCs ([71])	I ([72]) III ([70])	↑	CX3CL1/CX3CR1 ([63])	–
CCL7	PAECs VSMCs THP-1 ([73])	lymphocyte ([72])	I ([74]) III ([75])	↑	CCL7/CCR7	–
CXCL9	Macrophage ([76])	PAECs ([77])	I ([78]) III ([79])	↑	TAT1/CXCL9/CXCR3 ([80])	apoptosis ([77])
CXCL10	Macrophage ([76])	PAECs ([77])	IV ([81])	↑	–	apoptosis ([77])

## Pathophysiological mechanisms of PAH: PANoptosis and peripheral inflammation

### Overview of PANoptosis in PAH

#### Crosstalk between apoptosis, necroptosis, and pyroptosis: the basis of PANoptosis

Apoptosis, necroptosis, and pyroptosis have been proven to play vital roles in PAH. Moreover, increasing evidence indicates that these three death modes are closely connected at the molecular mechanism level.

caspase-8 (casp-8) was the first to be considered as the primary evidence of crosstalk among these three types of cell death [82]. To date, casp-8 is regarded as a protease that possesses both pro-death and pro-survival dual functions [83]. It was initially recognized as the initiator of extrinsic apoptosis in response to the activation of death receptors, such as Fas and Tumor Necrosis Factor (TNF) [84]. Subsequently, studies have found that casp-8 is also closely related to necroptosis. Newton found that casp-8 can lead to necrosome split by cleaving the Asp325 residue of Receptor Interacting Serine/Threonine Kinase (RIPK) 1, which in turn prevents the induction of necroptosis [85]. More evidence confirms that once casp-8 is inactivated or inhibited, it causes RIPK3 and Mixed Lineage Kinase Domain-Like (MLKL) dependent embryonic lethality in mice [83]. Subsequent studies have confirmed that casp-8/Fas-associating protein with a novel death domain (FADD) can inhibit the spontaneous activity of the RIPK3-MLKL pathway by influencing cyclic GMP-AMP synthase/stimulator of Interferon Genes/TANK binding kinase 1 (cGAS/STING/TBK1) and the positive feedback loop of Z-DNA binding protein 1 (ZBP1) [86]. Simultaneously, casp-8 dependent inflammatory responses are regulated by FADD and necroptosis [87]. It has been reported that casp-8 can also induce the transition from apoptosis to pyroptosis. Recent research shows that casp-8 cleavage of the D333 site in RIPK3 restricts the NLR family pyrin domain-containing (NLRP3) inflammasome-mediated pyroptosis and IL-1β secretion, while not inhibiting necroptosis [88]. Notably, Muendlein demonstrated that casp-8 can induce apoptosis-associated speck-like protein containing a CARD (ASC) oligomerization of NLRP3 inflammasom in the absence of casp-1/3/7 and BH3 interacting domain death agonist protein (BID), thereby activating casp-1-mediated pyroptosis and IL-1β release [89, 90]. These results indicate that casp-8 exhibits considerable plasticity in cell death pathways. Furthermore, recent studies have reported that when endothelial TGF-β-activated kinase 1 (TAK1) is inhibited or stimulated by lipopolysaccharides (LPS), casp-8 can directly cleave GSDMD and promote IL-1β expression through dimerization, self-processing, and activity upregulation [82, 89]. Casp-8 not only acts on the classical pyroptosis pathway but also affects the non-classical pathways mediated by GSDMC and GSDME. Upon various stimuli (such as metabolites and hypoxia), casp-8 specifically cleaves GSDMC and produces the GSDMC N-terminal domain, which causes membrane rupture and induces pyroptosis [91, 92]. Moreover, TNF-α/casp-8/casp-3/GSDME signal transduction mediated cell pyroptosis was found to promote muscular atrophy in the skeletal muscle cells of aged mice [93].

RIPK1 is currently recognized as a main node in the interaction between the three types of cell death. Initially, the focus was primarily on the mechanisms of necroptosis. It is now generally accepted that upon stimulation by death receptors such as TNF-R1, DR4/5, and Toll Like Receptor (TLR) 3/4, RIPK1 can bind to RIPK3, leading to the phosphorylation and activation of MLKL, which ultimately forms necrotic bodies and induces necroptosis [94]. Recent studies have revealed that RIPK1 also participates in pyroptosis and apoptosis in different forms. Numerous studies have confirmed when the Ser166 site of RIPK1 is phosphorylated after stimulation by Smac-mimics, 5Z-7 (TAK1 inhibitor), complex I (composed of TRADD, RIPK1 and E3 ubiquitin ligase) is degraded to mediate RIPK1-dependent apoptosis through complex IIb (also called ripoptosome, composed of RIPK1, TNF Receptor-Associated Death Domain (FADD) and casp-8) [95–97]. Studies have shown that the Asp325 site of RIPK1 is particularly important for the formation of TNF-responsive ripoptosomes and their concomitant manner of death [98]. Subsequent studies have verified that the C-terminally truncated version of ZBP1 (ZBP1ca) can induce RIPK3-dependent necroptosis and activate RIPK1-dependent apoptosis to a limited extent [99]. It has been reported that when infected with Yersinia, TLR/TNFR is activated and forms a FADD/RIPK1 complex to activate casp-8, thereby triggering pyroptosis mediated by GSDMD in macrophages and GSDME in neutrophils respectively [100, 101]. Meanwhile, glucose deprivation or activation of activated protein kinases K (AMPK), induced by reduced glucose levels within macrophages, can phosphorylate the S321 site of RIPK1 and promote the AMPK-RIPK1 cascade to inhibit pyroptosis [102]. Subsequent researches have found that RIPK1-dependent pyroptosis in these two immune cells is differentially regulated by immune signal transduction. Compared with neutrophils, which requires TNFR1 signal transduction to drive pyroptosis, macrophages rely on both TNFR1 and TLR4-TIR domain-containing adapter inducing IFN-β (TRIF) signaling pathways to trigger pyroptosis [103]. According to reports, non-classical autophosphorylation of RIPK1 at threonine 169 (T169) is essential for casp-8-mediated pyroptosis [104].

**Fig. 2:**
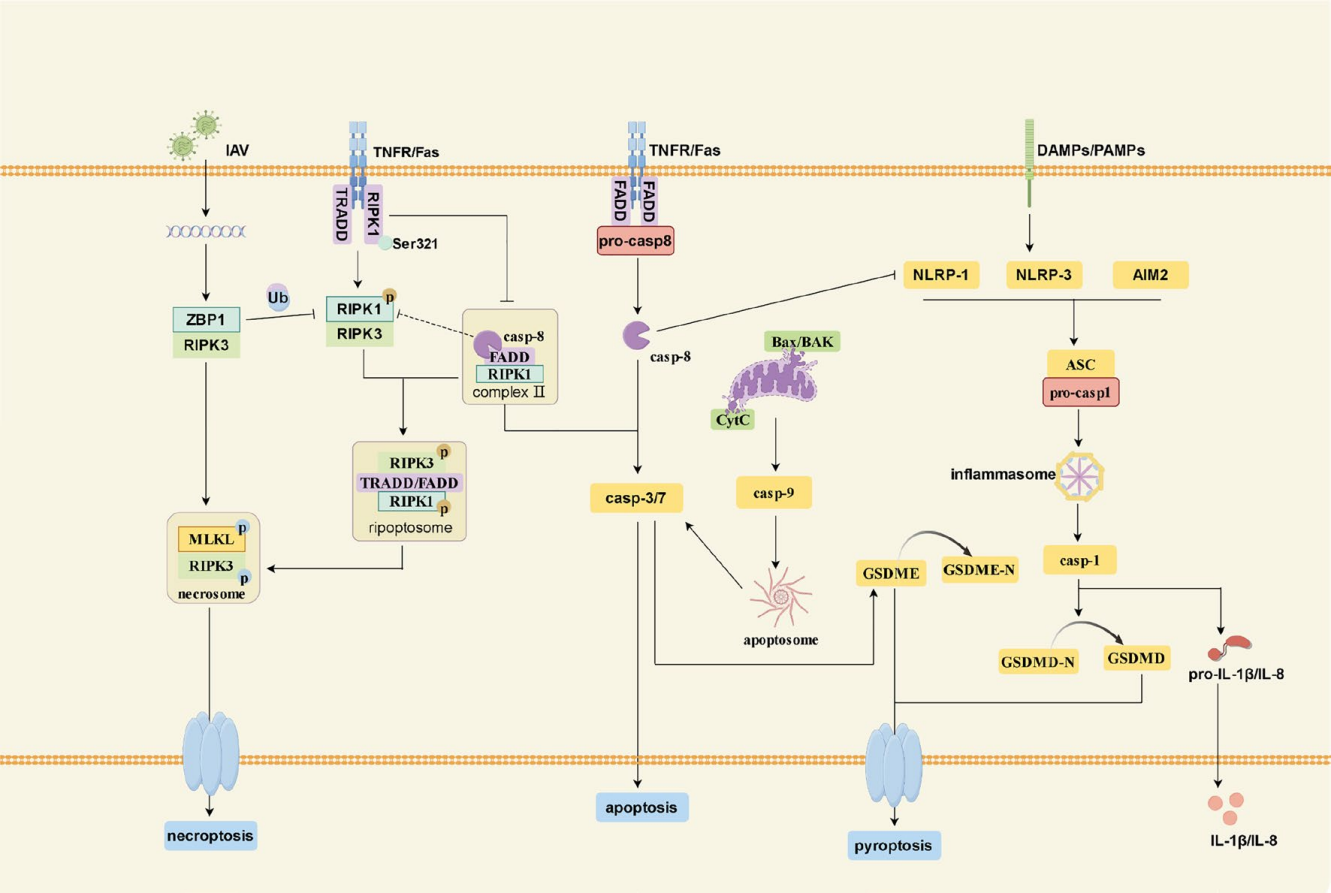
Crosstalk between apoptosis, necroptosis, and pyroptosis at the molecular level. Pyroptosis is primarily mediated by inflammasomes, which ultimately lead to the cleavage of GSDMD and the release of IL-1β and IL-8. Apoptosis is activated by casp-3/7, and its activation can be induced by the apoptosome formed through Bax/BAK/Cyt or stimulated by FADD/casp-8. It can also mediate cleavage of GSDME, triggering pyroptosis. Necroptosis is mainly mediated by necrosomes that are composed of RIPK3 and MLKL. Additionally, FADD/casp-8 can form complex II by binding to RIPK1.When casp-8 is inhibited, complex II binds to RIPK3 to form necrosomes, thereby inducing necroptosis. This diagram was drawn by Figdraw (www.figdraw.com)

The aforementioned evidence confirms the interactive relationship between apoptosis, necroptosis, and pyroptosis. When the regulatory effect of a specific factor is inhibited or interfered with, other pathways can play compensatory roles. Importantly, these three modes of cell death can coexist under pathological conditions. Based on repeatedly validated evidence, it can be speculated that PANoptosis exists in PAH (Fig. 2).

#### Molecular mechanism of PANoptosis

Since PANoptosis exhibits the fundamental characteristics of the three aforementioned modes of cell death in terms of molecular mechanisms and morphological features, it has been formally designated as "PANoptosis", where "P, A, N" represent pyroptosis, apoptosis, and necroptosis, respectively [20, 105]. It is an inflammatory lytic cell death pathway driven by caspases and RIPKs, and is regulated by the PANoptosome [106]. The PANoptosome is essential for initiating cell death and sensing stimuli such as pathogen-associated molecular patterns (PAMPs) and damage-associated molecular patterns (DAMPs) [105]. It includes five types: ZBP1-, Absent in melanoma 2 (AIM2)-, RIPK1-, NLRP12- and NLRC5-PANoptosome [107]. Similar to most protein complexes, the PANoptosome primarily consists of three components: the sensing domain, the assembly domain, and the catalytic domain [105]. The catalytic domain is composed of various catalytic effectors, such as casp-1/8, RIPK3 and RIPK1 [105]. The assembly domain includes adaptor proteins with caspase recruitment domains, including FADD, ASC, and NLRP3 [21]. The sensing domain mainly encompasses ZBP1- [108], AIM2- [109], RIPK1- [101], NLRP12- [110] or NLRC5- [111] adapters. It can activate different types of PANoptosomes in response to different pathogenic microorganisms or other stress conditions. These domains play a important role in forming the PANoptosome through homotypic(RIP homotypic interaction motifs (RHIM-RHIM), DD-DD, DED-DED, CARD-CARD) and heterotypic (DED-PYRIN) interactions between proteins [112].

#### ZBP1-PANoptosome

**Fig. 3:**
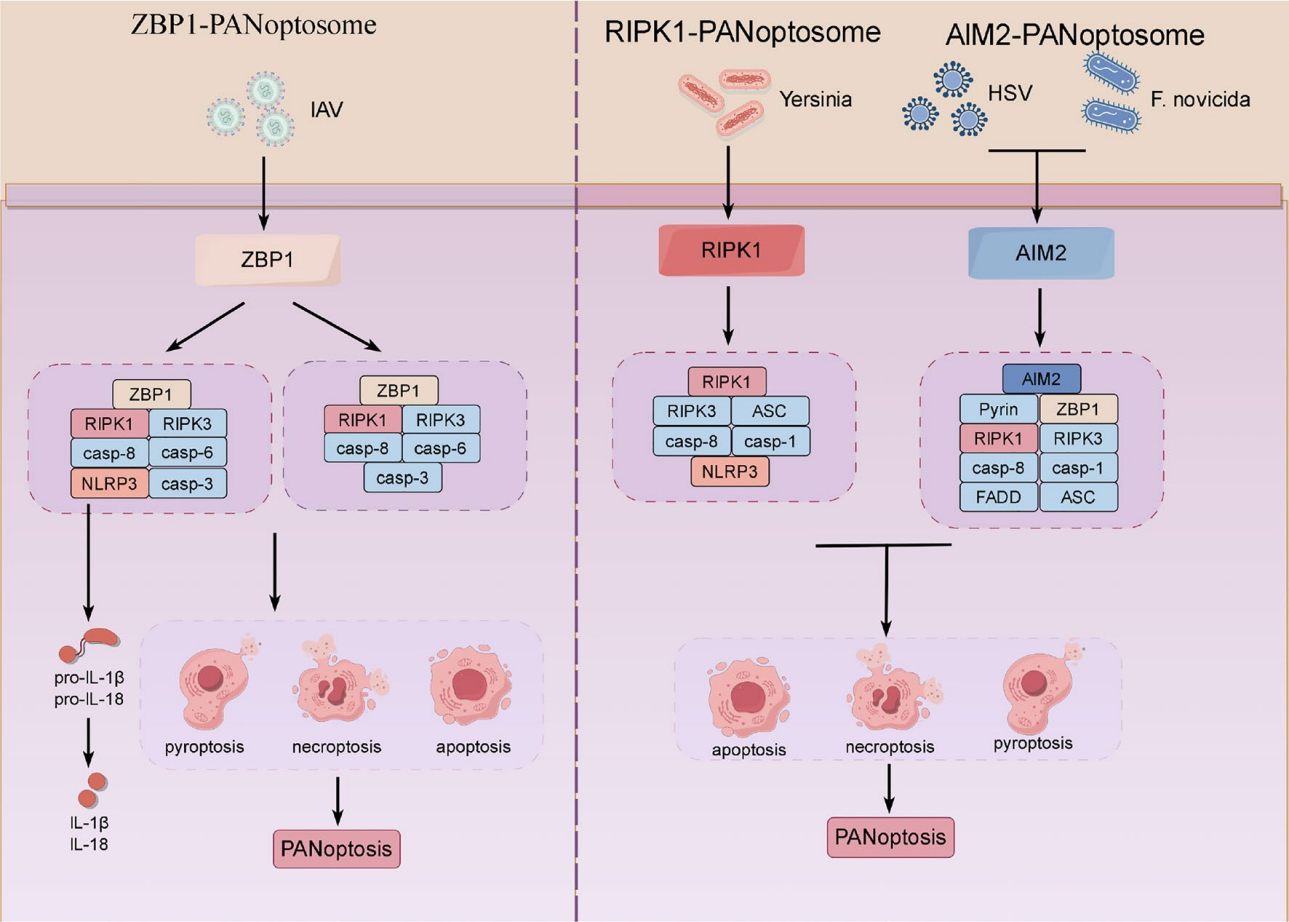
Composition of the PANoptosome. When the ZBP1 PANoptosome is activated by IAV stimulation, it induces the occurrence of PANoptosis. It can be divided into two categories: one is composed of ZBP1, RIPK1, RIPK3, casp-8/6/3, and NLRP3, where NLRP3 mediates the release of IL-1β/18; the other category lacks NLRP3 and is unable to produce inflammatory factors. The RIPK1 PANoptosome consists of ASC, RIPK3, RIPK1, casp-1/8, and NLRP3, and is activated by Yersinia. The AIM2 PANoptosome is composed of AIM2, Pyrin, ZBP1, ASC, casp-1/8, RIPK3, RIPK1, and FADD, and is activated by HSV-1 and F.novicida. This diagram was drawn by Figdraw (www.figdraw.com)

ZBP1 PANoptosis was first discovered during influenza A virus (IAV) infection [113]. It is mainly composed of ZBP1, NLRP3, casp-8/6/3, RIPK1 and RIPK3 [107] (Fig. 3). ZBP1 is a cytoplasmic Z-nucleic acid sensor that contains two Z-nucleic acid binding domains (Zα domains) at the N-terminus: Zα-1 and Zα-2 [108]. These two domains possess RHIM1 and RHIM2 in the protein sequence [108], with RHIM1 capable of binding to RIPK3 to induce necroptosis [114]. Interestingly, Zα-2 has been found to be closely related to death. It regulates death in conjunction with RHIM1 [115, 116] and senses nucleic acids [117], acting as a main innate immune sensor for Z-RNAs. However, the specific functions of Zα-1 and RHIM1 require further investigation. It has been reported that ZBP1 recruits casp-8 to form the ZBP1/RIPK3/casp-8 signaling scaffold after binding to RIPK3 [20], and then successively activates the three death modes and PANoptosis. The NLRP3 inflammasome triggers the production of IL-1β and IL-18 during PANoptosis, which is unrelated to cell death [118]. This indicates that NLRP3 is not essential in this complex. When the ZBP1 PANoptosome lacks the NLRP3 inflammasome, the maturation of IL-1β and IL-18 is reduced or inhibited, but cell death occurs due to compensatory functions of molecules such as casp-3/6 [118].

#### RIPK1-PANoptosome

The RIPK1 PANoptosome is composed of ASC, RIPK3, RIPK1, casp-1/8, and NLRP3 [107] (Fig. 3). RIPK1, as the inaugural member of the receptor-interacting serine/threonine kinase family, is a vital regulatory factor in mediating cellular inflammation [119]. Initially, ZBP1-independent PANoptosis was discovered by Malireddi during Yersinia infection in 2020 [120]. This study found that RIPK1 expression deficiency resulted in apoptosis and pyroptosis reduction, but necroptosis increased in gene knockout mouse models, whereas other proteins did not exhibit this [120]. This indicates that RIPK1 is a critical factor driving PANoptosis. It has been reported that TAK1 is essential for PANoptosome regulation [119, 121]. A previous study has shown that Yersinia infection can inhibit TAK1 to phosphorylate RIPK1 and activate RIPK1-PANoptosis [120].

#### AIM2-PANoptosome

AIM2 PANoptosis was identified during HSV 1 and F.novicida infections in 2021 [122]. It is composed of AIM2, Pyrin, ZBP1, ASC, casp-1, casp-8, RIPK3, RIPK1 and FADD [122] (Fig. 3). AIM2 is a DNA sensor consisting of an N-terminal pyrin domain and C-terminal HIN domain [107]. It interacts with Pyrin and ZBP1 through ASC to form a multiprotein complex [122]. Furthermore, it has been reported that the knockout of AIM2 reduces the expression levels of Pyrin and ZBP1, indicating that AIM2 acts as an upstream regulatory factor of Pyrin and ZBP1 to regulate the assembly and activation of the PANoptosome [122]. Subsequently, the interaction of ASC with AIM2, Pyrin, ZBP1, casp-1/8, RIPK3, RIPK1, and FADD was observed by immunoprecipitation [122].

#### NLRP12-PANoptosome

**Fig. 4:**
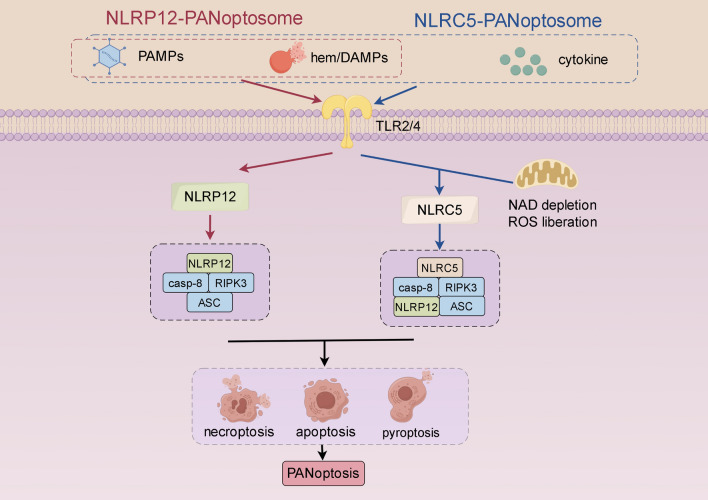
Composition of the PANoptosome. The NLRP12 PANoptosome is induced by TLR2/4 and is composed of NLRP12, ASC, casp-8, and RIPK3. The NLRC5 PANoptosome is induced after stimulation by TLR2/4 or mitochondrial damage, and consists of NLRC5, NLRP12, NLRP3, casp-8, ASC, and RIPK3. This diagram was drawn by Figdraw (www.figdraw.com)

NLRP-12 PANoptosis was first discovered by Sundaram in 2023 as a new protein complex that responds to heme and PAMPs [110]. It consists of NLRP12, ASC, casp-8 and RIPK3 [123] (Fig. 4). NLRP12, as a member of the NLR family, is involved in the regulation of inflammatory signaling in response to infection and cell stress and can form inflammasomes composed of various oligomeric proteins [124]. A recent study has revealed that organs activate TLR-2/4-MyD88 to specifically induce the generation of the NLRP12 PANoptosome and induce the maturation of IL-1β and IL-18 after stimulation with heme and PAMPs or two different PAMPs [110]. However, whether there are other synergistic effects or other signaling pathways that cause activate PANoptosome remains to be resolved and requires further research and exploration.

#### NLRC5-PANoptosome

The NLRC5 PANoptosome is a novel assembly discovered in 2024 that consists of NLRC5, NLRP12, NLRP3, casp-8, ASC and RIPK3 [111] (Fig. 4). As an innate immune sensor, NLRC5 responds to ligand-driven cell death [111]. To determine the relationship between NLRC5 and NLRP12, Sundaram utilized double-knockout mice and found that the absence of NLRC5 significantly increased death in response to heme and PAMPs stimulation [111]. This indicated that NLRC5 acts as a sensor when exposed to a specific ligand. Furthermore, immunoprecipitation revealed that TLR-2/4 and NAM are significant factors driving the NLRC5 PANoptosome [111].

#### PANoptosis in PAH

Current research shows that apoptosis, pyroptosis, and necroptosis can promote the formation of vascular remodeling by mediating the phenotypic and activity transformation of pulmonary vascular cells [125]. And it is evident that there is crosstalk among these three modes of cell death. Therefore, it can be inferred that PANoptosis is involved in the formation of vascular remodeling.

#### Apoptosis in PAH

Apoptosis resistance represents a fundamental phenotypic transformation of pulmonary vascular cells. Pulmonary arterial endothelial cells (PAECs) have been reported to show a pro-apoptotic states in the early stage of PAH, and transform into an apoptosis-resistant phenotype in the later stages of the disease [125, 126]. This not only indicates that the pathological mechanisms of PAH are complex, but also reveals the temporal heterogeneity of the abnormal pathological state of PAECs. It is generally believed that apoptosis of PAECs in the early stage is related to the lack of bone morphogenetic protein receptor type 2 (BMPR-2) signal transduction or the activation of casp-3 by the pro-apoptotic factor programmed cell death-4 (PDCD4) induced by epigenetic modifications [127]. With disease deterioration, the proliferation and anti-apoptotic phenotype of PAECs mainly depends on the activation of signal transduction and activator of transcription 3 (STAT3) [128]. Interestingly, both the proliferative and apoptotic phenotypes of PAECs can promote vascular remodeling [15], but the specific reasons remain unclear. However, pulmonary arterial smooth muscle cells (PASMCs) display an apoptosis-resistant phenotype following the onset of the disease [129]. Studies have shown that heterogeneous nuclear ribonucleoprotein A2B1 (HNRNPA2B1) serves as the main coordinating protein for the proliferative/anti-apoptotic phenotype of PASMCs [130]. Interestingly, GATA6-deficient PAECs have been found to increase GATA6-dependent proliferation and the anti-apoptotic phenotype of PASMCs [131]. Subsequently, Zhang discovered that the activation of autophagy under hypoxic conditions induces the proliferation of PAECs and apoptosis of microvascular ECs (MVECs) and leads to the replacement of MVECs by PAECs, finally providing a vascular microenvironment that promotes the proliferation and migration of PASMCs, ultimately driving the development of PAH [132]. Thus, PAECs can establish communication with PASMCs and induce apoptosis resistance.

#### Pyroptosis in PAH

Pyroptosis plays a significant role in pathophysiological changes in PAH and has a substantial impact on right ventricular hypertrophy [27, 133]. Current studies have demonstrated that post-translational modifications [134], non-coding RNAs (ncRNAs) [135, 136] and gut microbiota metabolites [137] can mediate pulmonary vascular cells to undergo pyroptosis to participate in vascular remodeling. Pyroptosis includes two pathways: the classical pathway mediated by casp-1 and the non-classical pathway mediated by casp-4/11 [8]. Casp-1 mediated pyroptosis is involved, to some extent, in vascular remodeling, such as BMPR2 signaling upregulator1 (BUR1) and GPR146 [134, 138]. Recent findings have found that casp-4/11/GSDME and BMPR2/casp-3/GSDME-mediated pyroptosis pathways also play important roles in the pathogenesis of PAH [139, 140]. Pulmonary vascular cells can mediate pyroptosis through signaling pathways such as prolyl hydroxylase domain-2 (PHD2)/hypoxia inducible factor-1α (HIF-1α) [135] and AMPK/NF-kappaB(NF-κB)/NLRP3 [137], thereby forming a positive feedback loop that exacerbates vascular remodeling. Interestingly, programmed death-ligand 1 (PD-L1) regulated by STAT1 can mediate casp-1 mediated pyroptosis in PASMCs to modulate pulmonary vascular fibrosis and accelerate the progression of PAH [134]. However, Wang found that PD-1/PD-L1 was downregulated by ubiquitination in hypoxic pulmonary hypertension (HPH), and this pathway could inhibit T helper 17 cell response and improve endothelial dysfunction [136]. Since pyroptosis is a form of cell death, further exploration is needed to understand how its activation ultimately leads to pathological changes, such as the proliferation of pulmonary vascular cells and cardiomyocytes, as well as hemodynamic alterations.

#### Necroptosis in PAH

Despite limited research on the mechanisms of necroptosis in PAH, it is widely believed that it exists in PAH and promotes vascular remodeling [7, 15]. Recently, Xiao et al. found that RIPK3-mediated necroptosis activates the TLR and NLR pathways to participate in PAH by activating DAMPs [141]. Subsequently, Jarabicová found that the mRNA expression levels of RIPK3 and MLKL increased in an MCT-induced PAH rat model [142]. It has been reported that high mobility group box 1 (HMGB1) is an important mediator of inflammation and vascular repair in PAH [143]. Later, Zemskova noted that the release of HMGB1 may be related to necroptosis [144]. These findings suggest that necroptosis is a potential mechanism mediating vascular remodeling and inflammation and the upregulation of pThr^231^/Ser^232−RIP3^ causes RV necroptosis in PAH. The expression level of RIPK3 in plasma was positively correlated with the Fulton index, and this increase was more significant in the late stages [142]. This indicates that RIPK3-mediated necroptosis is correlated with the prognosis of PAH. Although necroptosis has been shown to be involved in the development of PAH, its specific mechanism of action requires further research and exploration.

#### The potential molecular mechanism of PANoptosis in PAH

**Fig. 5:**
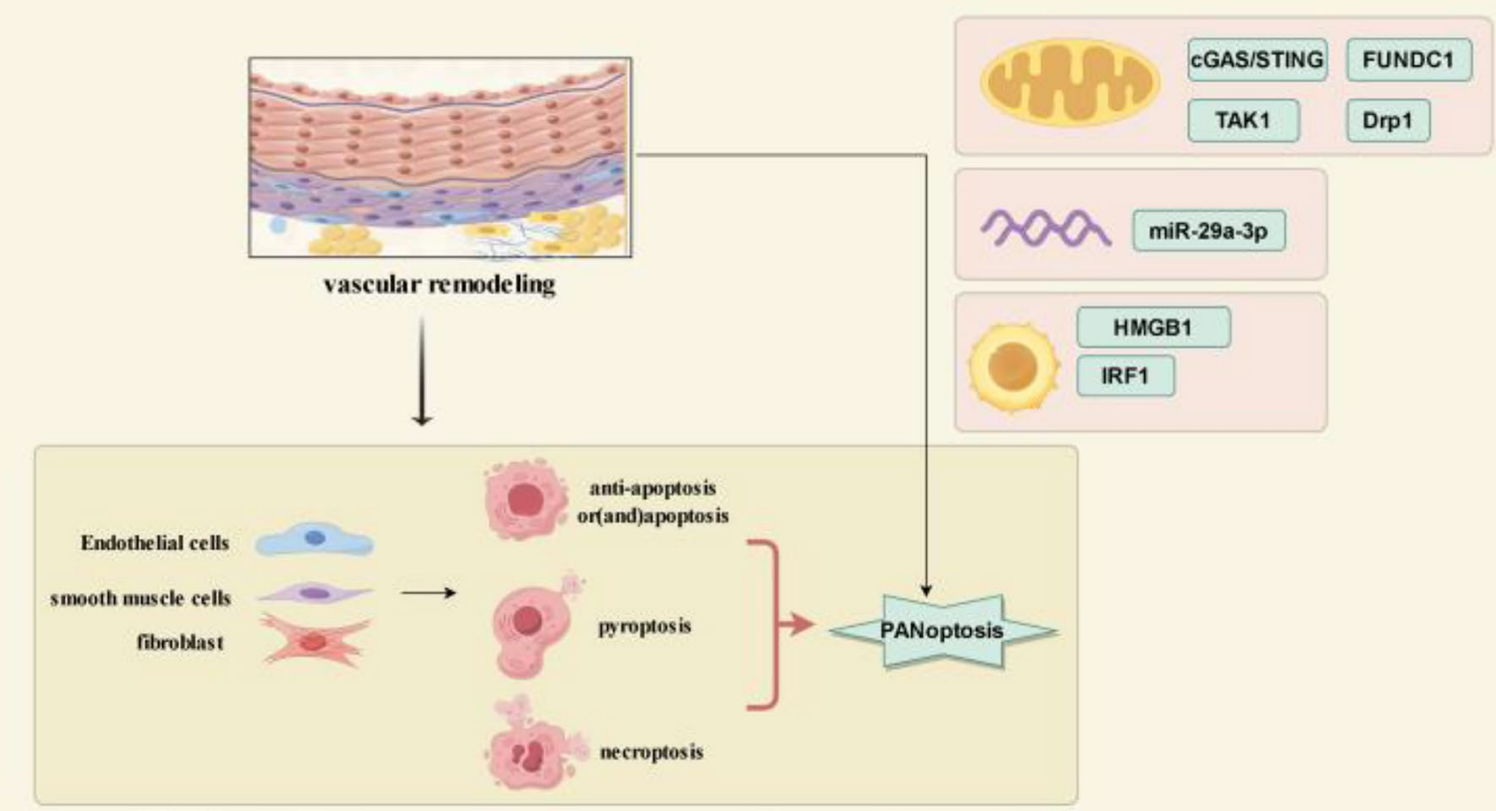
Relationship between PANoptosis and PAH. The activation of abnormal cell death in pulmonary vascular cells (ECs, SMCs, and fibroblasts) is one of the causes of vascular remodeling. Typical deaths include apoptosis resistance/apoptosis, pyroptosis, and necroptosis, whereas PANoptosis exhibits morphological changes characteristic of the above three. Furthermore, vascular remodeling can be associated with PANoptosis through the regulation of certain factors, including mitochondria-related components such as cGAS/STING, TAK1, Drp1, and FUNDC1, as well as immune-related factors such as HMGB1, Interferon regulatory factor-1 (IRF1), and miR-29a-3p. This diagram was drawn by Figdraw (www.figdraw.com)

It has been reported that STING, as a radical target of PAH, can induce PANoptosis [145, 146]. And Meng found that the DNA damage/cGAS/STING pathway can exacerbate PAH by promoting cell proliferation and phenotypic transformation [147]. It has been found that this pathway is activated by mitochondrial DNA (mtDNA) cytoplasmic escape and induces PANoptosis in response to intermittent hypoxia [148]. Moreover, certain mechanistic targets in PAH can induce or inhibit PANoptosis to some extent. Studies have shown that FUNDC1 not only regulates mitochondrial dynamics and energy metabolism to improve PAH [149], but also inhibit the activation of PANoptosis through interaction with mitochondrial Tu translation elongation factor (TUFM) [150]. Furthermore, factors such as dynamin-related protein 1 (DRP1), TAK1, and HMGB1 are involved in vascular remodeling [151–153], and PANoptosis is also related to DRP1-mediated mitochondrial fission and passive release of HGMB1 [154–156]. Interestingly, current research shows that TAK1 deficiency causes RIPK1-mediated PANoptosis by regulating mitochondrial redox balance [157], suggesting that the mechanism of TAK1 involvement in vascular remodeling and PANoptosis may not be consistent. Thus, it can be inferred that PANoptosis may occur in PAH, and impaired mitochondrial quality could be one of the driving factors for the occurrence of PANoptosis. Additionally, Luo confirmed that miR-29a-3p, regulated by HIF-1α and SMAD family member 3 (Smad3), could significantly improve HPH and right ventricular hypertrophy [158]. Recent studies have found that it can target TNFR1 to inhibit the occurrence of PANoptosis [159]. This suggests that miRNAs may also be one of the factors driving the occurrence of PANoptosis in PAH (Fig. 5).

### Overview of perivascular inflammation in PAH

#### Cytokines

##### Interleukin-1 (IL-1)

Cytokines related to the IL-1 family play vital roles in inflammatory responses and have become central mediators in numerous diseases [160, 161]. It has been reported that the increased biological activity of IL-1 is associated with MCT-induced inflammation and PAH progression [162, 163]. Subsequent research revealed that the use of IL-1 antagonists can prevent the increase in pulmonary vascular resistance and pulmonary vascular remodeling in certain forms of PAH [35]. The IL-1 family contains 11 members, including IL-1α, IL-1β, and IL-1Ra [163], of which IL-1β is generally considered a radical inflammatory mediator involved in pathological pulmonary vascular remodeling [32, 163]. It has been reported that the uptake and translation of mRNA in activated HPAECs are higher following stimulation by TGF-β1 and IL-1β in PAH [164]. Subsequent studies found that IL-1β can participate in vascular remodeling and inflammatory responses by activating corresponding pathways, such as IL-1R1/MyD88 [40]. More importantly, IL-1β has been confirmed to be involved in various types of PAH (as detailed in Table 1). Interestingly, Agrawal discovered that macrophage-mediated IL-1β partially regulates cardiac remodeling in an animal model of heart failure with preserved ejection fraction (HFpEF)-PH [32].This indicates that IL-1β from myeloid cells may be associated with the prognosis of PAH.

##### Interleukin-6 (IL-6)

IL-6 is a widely distributed pluripotent cytokines and serves as an independent risk factor in the pathogenesis of PAH. To date, numerous studies have confirmed that IL-6 can activate various signaling pathways that drive the progression of PAH, such as CD4 + T cell/IL-6/glycoprotein 130 (gp130) [165], IRF-4/IL-6/ C-X3-C-Motif Receptor 1 (CX3CR1) [166], and IL-6/Forkhead box protein O1 (FoxO1) [167]. In addition, IL-6 can mediate the mobilization of neutrophils from the bone marrow in PAH, and this mobilization depends on the expression of CX3CR1 in neutrophils [166]. Interestingly, however, progesterone can reverse the adverse effects of IL-6 in PAH and block pSmad1-Id1/2 axis in IL-6-incubated PASMCs to improve vascular remodeling [168]. Recently, Simpson analyzed data, which was from the PAH biorepository of the National Institutes of Health and the National Heart, Lung, and Blood Institute, and found that IL-6 is upregulated to varying degrees in different PAH subtypes, with the highest levels observed in connective tissue disease-related PAH (CTD-PAH) and portopulmonary hypertension [169]. Reports indicate that IL-6 levels are independently associated with right ventricular (RV) function and RV-pulmonary artery coupling in PAH. That is, with equal PAH levels, patients with higher IL-6 expression have relatively worse right heart function [170]. This indicates that IL-6 can be used as a biomarker to assess the severity and prognosis of PAH [45, 171]. It is worth noting that Woolf used Mendelian randomization analysis and found that there was no causal relationship between IL-6 and PAH risk [172]. However, Takeyasu proposed that an IL-6 ≥ 2.73 pg/mL could serve as a detection standard for identifying PAH with an “inflammatory response phenotype” [173].

##### Interleukin-18 (IL-18)

IL-18 is a member of the IL-1 family of cytokines and can display pleiotropic effects depending on the cytokine environment [174]. Vascular remodeling is closely related to increased IL-18 expression and the persistent inflammatory environment [56]. Subsequent research has indicated a strong correlation between IL-18 and PAH, and its maturation depends on casp-1 [54]. Hillestad later discovered that IL-18 is associated with left ventricular diastolic dysfunction during alveolar hypoxia [175]. However, Bruns found that knockout of the IL-18 gene alone did not protect mice from right ventricular hypertrophy induced by HPH, suggesting that there may be parallel activated inflammatory pathways that play a compensatory role [176].

#### Chemokines

During the inflammatory response in PAH, signal transduction between chemokines and their corresponding receptors plays a crucial role in promoting the infiltration of immune cells into the lungs [177]. Studies have shown that monocyte chemoattractant protein-1 (MCP-1, also known as CCL2) is involved in the progression of various types of PAH (Table 1). The expression of MCP-1 was negatively correlated with disease duration [62]. Florentin et al. found that although MCP-1 is involved in lung inflammation and pulmonary vascular remodeling, it has no significant effect on hemodynamic changes [178]. Furthermore, the CCL2/CCR2 axis is one of the important signaling mechanisms involved in the pathological stages of PAH [46]. Studies have shown crosstalk between MCP-1 and CCL5, and this interaction is essential for the collaboration between macrophages and PASMCs [179]. CCL5 deficiency can reverse obstructive changes in the pulmonary arteries by restoring BMPR2 signaling [69]. Moreover, elevated CCL5 expression are associated with deterioration of cardiac function and poor prognosis [74]. CX3CL1 exists as a cell adhesion molecule or chemokine on ECs [180], which not only responds to the inflammatory response but also responds to the crosstalk between ECs and SMCs during hypoxia through the CX3CL1/CX3CR1 axis [70]. Specifically, CX3CL1 secreted by ECs triggers phenotypic changes in PASMCs. Studies have found that CCL7 can exert effects on PAH by mediating the lymphocyte secretion of CX3CL1 [72].

Current research confirms that numerous chemokines and cytokines are involved in the occurrence and development of PAH, and crosstalk among them, which is closely related to the severity and prognosis of the disease. More importantly, some factors were closely associated with various modes of death, as detailed in Table 1.

## Interplay between PANoptosis and perivascular inflammation in PAH

It is generally believed that PANoptosis is a unique inflammatory death mode independent of the above three death modes. It can be activated by stimuli such as viral or bacterial infections and cytokine storms [123]. Therefore, it is speculated that PANoptosis is closely related to inflammation and may be involved in pathophysiological changes associated with PAH.

Pyroptosis and Perivascular Inflammation in PAH.

Pyroptosis is now commonly recognized as a form of secondary necrosis and a well-recognized inflammatory death that serves to defend against pathogens [181]. It causes the release of inflammatory factors such as IL-1β, IL-18, and IL-6, and recruits neutrophils and macrophages to exacerbate the inflammatory response and vascular remodeling [55]. NLRP3/casp-1/IL-1β-mediated pyroptosis is one of the driving factors of local inflammation in PAH [133, 182]. It is generally noted that IL-21 produced by follicular helper T cells (T_FH_) promotes the proliferation and pyroptosis of PASMCs in HPH, while conversely, the selective CX3CR1 antagonist AZD8797 inhibits PASMCs pyroptosis [183]. Thus, the initiation of pyroptosis and inflammation is significantly associated with disease mechanisms.

### Apoptosis and inflammation in PAH

Research has found that IL-1β mediated by pyroptosis can induce apoptosis [184–186]. Previous studies have shown that IL-6 can induce apoptosis resistance in PASMCs through different signal transduction pathways, thereby promoting vascular remodeling in PAH, such as IL-6/STAT3/Kruppel-like factor 5 (KLF5) [187] and IL-6/bromodomain protein 4 (BRD4) [188]. M2b macrophages, as central effector cells in the local inflammation of PAH, can promote apoptosis and migration of PASMCs by regulating the expression of Bcl-2 family proteins through the inhibition of the PI3K/Akt/FoxO3a [189]. In addition, Pan found that sulforaphane (SFN) extracted from plants can reduce the expression levels of inflammatory factors such as TNF-α, IL-6 and NF-κB, thereby promoting PASMCs apoptosis and inhibiting pulmonary microvascular ECs apoptosis, ultimately improving vascular remodeling [190]. Interestingly, PAECs apoptosis promotes vascular remodeling in PAH [191]. Further research confirms that once hypoxic stimulation increases, it activates the NF-κB signaling pathway and promotes the upregulation of chemokines (cytochrome P450 family 1 subfamily A member 1 (CYP1A1) and cytochrome P450 family 1 subfamily B member 1 (CYP1B1)) and inflammatory factors, such as IL-1β and IL-6, eventually leading to PAECs apoptosis [43]. Studies have also shown that 15-hydroxyeicosatetraenoic acid (15-HETE) can activate T cell-dependent PAECs apoptosis mediated by oxidized lipids to induce PAH [192]. In summary, disordered inflammation and apoptosis are closely related to the disease, and their interactions have different mechanistic effects on pulmonary vascular cells.

### Necroptosis and inflammation in PAH

Necroptosis is now considered an inflammatory form of death, so scholars speculate that its abnormal activation may also be one of the mechanisms that trigger inflammation [193]. In addition, RIPK1 kinase can act as a transcriptional co-regulator in the nucleus and directly regulate and participate in the transcription of specific genes that mediate inflammatory responses [194]. RIPK1 inhibitors (Nec-1) can inhibit necroptosis and downregulate the expression of IL-1β, IL-6, and TNF-α in certain inflammatory bowel diseases [195, 196]. These results indicated that necroptosis is closely related to inflammation and is involved in the occurrence and development of various inflammatory diseases. Although there is currently limited research on the specific mechanisms of necroptosis in PAH, scholars have confirmed and summarized that necroptosis is an important death mode involved in vascular remodeling [15, 23, 141]. Subsequently, Xiao confirmed that RIPK3-mediated necroptosis participates in the production of DAMPs in MCT-induced PAH, and enriches TLR and NLR pathways, and increases inflammation levels [141]. Given that inflammation also plays a major role in vascular remodeling in PAH, it is reasonable to speculate that necroptosis may exacerbate inflammation progression, further promoting vascular remodeling in PAH.

### PANoptosis and inflammation in PAH

IRF1 is an immunoregulatory factor and an important molecule that mediates inflammation and cell death. Existing studies have noted that it is not only a core factor of PAH pathological changes [197] but can also activate ZBP1-, AIM2-, RIPK1-, and NLRP12-PANoptosis [198]. As an important mediator secreted by immune cells, HMGB1 can cause an increase of IL-6 and CXCL8 in PASMCs and PAECs in PAH, so it is widely regarded as an inflammatory driver of PAH [153]. at the same time, it also mediates the occurrence of PANoptosis [155].

## Conclusion and future perspective

PAH, a progressive malignant disease driven by various heterogeneous causes, imposes a significant burden on the world, particularly in low- and middle-income countries. Currently, available pharmacological treatments in clinical practice mainly focus on three pathways: prostacyclin, endothelin, and nitric oxide (NO) pathways. Their primary function is to dilate blood vessels to reduce pulmonary artery pressure, which can alleviate or relieve the symptoms or signs of PAH, and the overall treatment outcomes do not meet the expected levels. Therefore, it is particularly important to transform innovative scientific knowledge into medical intervention measures. With an in-depth study and understanding of the pathophysiology of PAH, and PCD and inflammatory responses have been found to be the main pathogenesis of PAH. Apoptosis/apoptosis resistance, necroptosis, and pyroptosis in PCD have emerged as closely intertwined with both the occurrence and prognosis of PAH. Existing studies have pointed out that PANoptosis can induce the occurrence of the above three death modes by mediating the PANoptosome, independent of any single mode of demise. PANoptosis serves as the basis for inflammation and immune responses; thus, PANoptosis and inflammation may play synergistic or cooperative roles in PAH.

The emergence of PANoptosis represents a compensatory or redundant effect within the pathological mechanisms of PAH; therefore, precise treatment can optimize its efficacy. Although the exploration of PANoptosis in PAH is currently limited, targeted therapy can be achieved by interfering with the components or upstream factors of the PANoptosome, such as NLRP3, the caspase family, and TAK1. Currently, some drugs (e.g. pirfenidone [199] and Nec-1 s [144]) have been shown to improve PAH, which also indicates that there may be changes in the treatment of PAH. Additionally, the presence of PANoptosis also suggests that there may be other uncharacterized death modes or regulatory factors in PAH. Therefore, this review provides strong evidence for the interplay between them, providing new ideas for subsequent exploration of the pathophysiological mechanisms and treatment methods of PAH.

## Limitations

Although certain drugs have been identified as improving PAH through the targeting of specific PANoptotic factors, several obstacles to their effectiveness and clinical application remain. a) As a whole, the use of these targeted drugs may non-specifically regulate other proteins, potentially resulting in unintended biological effects or side effects on other organs or tissues. b) Given that PANoptosis is closely associated with specific inflammatory processes, certain drugs may impact immune function and potentially lead to immune disorders. c) PAH frequently coexists with underlying diseases that may exhibit functional differences across various organs and conditions; thus, controlling the dosage and administration of drugs may be necessary. d) Significant differences may also exist in the dosage and administration of drugs across species, resulting in discrepancies in their application different species.

## Data Availability

No datasets were generated or analysed during the current study.
